# Weight Loss Treatment and Longitudinal Weight Change Among Primary Care Patients With Obesity

**DOI:** 10.1001/jamanetworkopen.2023.56183

**Published:** 2024-02-15

**Authors:** James Henderson, Anne P. Ehlers, Joyce M. Lee, Andrew T. Kraftson, Kenneth Piehl, Caroline R. Richardson, Dina H. Griauzde

**Affiliations:** 1 Department of Internal Medicine, University of Michigan, Ann Arbor; 2 Institute for Healthcare Policy and Innovation, University of Michigan, Ann Arbor; 3 Department of Surgery, University of Michigan, Ann Arbor; 4 Veteran Affairs Ann Arbor Healthcare System, Ann Arbor, Michigan; 5 Department of Pediatrics, University of Michigan, Ann Arbor; 6 Department of Obstetrics and Gynecology, University of Michigan, Ann Arbor; 7 Department of Family Medicine, Brown University, Providence, Rhode Island

## Abstract

**Importance:**

Among individuals with obesity, 5% or greater weight loss can improve health. Weight management treatments (WMT) include nutrition counseling, very low-calorie meal replacement (MR), antiobesity medications (AOM), and bariatric surgery; however, little is known about how these WMT are associated with weight change among individual patients and populations.

**Objective:**

To characterize weight status and WMT use among primary care patients and assess associations between WMT and weight trajectories.

**Design, Setting, and Participants:**

Retrospective, population-based cohort study of primary care patients from 1 academic health system in Michigan between October 2015 and March 2020 using cross-sectional analysis to compare obesity prevalence and WMT utilization. For patients with obesity and WMT exposure or matched controls, a multistate Markov model assessing associations between WMT and longitudinal weight status trajectories was used. Data were analyzed from October 2021 to October 2023.

**Exposures:**

Cross-sectional exposure was year: 2017 or 2019. Trajectory analysis exposures were WMT: nutrition counseling, MR, AOM, and bariatric surgery.

**Main Outcomes and Measures:**

Cross-sectional analysis compared mean body mass index (BMI), obesity prevalence, and, among patients with obesity, prospective WMT use. The trajectory analysis examined longitudinal weight status using thresholds of ±5% and 10% of baseline weight with primary outcomes being the 1-year probabilities of 5% or greater weight loss for each WMT.

**Results:**

Adult patients (146 959 participants) consisted of 83 636 female participants (56.9%); 8940 (6.1%) were Asian, 14 560 (9.9%) were Black, and 116 664 (79.4%) were White. Patients had a mean (SD) age of 49.6 (17.7) years and mean (SD) BMI of 29.2 (7.2). Among 138 682 patients, prevalence of obesity increased from 39.2% in 2017 to 40.7% in 2019; WMT use among patients with obesity increased from 5.3% to 7.1% (difference: 1.7%; 95% CI, 1.3%-2.2%). In a multistate model (10 180 patients; 33 549 patient-years), the 1-year probability of 5% or greater weight loss without WMT exposure was 15.6% (95% CI, 14.3%-16.5%) at reference covariates. In contrast, the probability of 5% or greater weight loss was more likely with year-long exposures to any WMT (nutrition counseling: 23.1%; 95% CI, 21.3%-25.1%; MR: 54.6%; 95% CI, 46.5%-61.2%; AOM: 27.8%; 95% CI, 25.0%-30.5%; bariatric surgery: 93.0%; 95% CI, 89.7%-95.0%).

**Conclusions and Relevance:**

In this cohort study of primary-care patients with obesity, all WMT increased the patient-level probability of achieving 5% or greater weight loss, but current rates of utilization are low and insufficient to reduce weight at the population level.

## Key Points

**Question:**

To what extent do weight management treatments (WMT) support 5% or greater weight loss among primary care patients and populations with obesity?

**Findings:**

In this cohort study of 146 959 participants, only 7.1% of primary care patients with obesity utilized WMT in 2019. Based on a multistate model examining weight trajectories among 10 180 patients with obesity, the 1-year probability of 5% or greater weight loss without WMT exposure was 15.6% and significantly increased with year-long exposures to any WMT, from 23.1% for nutrition counseling to 93.0% for bariatric surgery.

**Meaning:**

These findings suggest WMT can support 5% or greater weight loss for individual patients, but low utilization limits their population-level potential.

## Teaser

This cohort study evaluates the extent to which weight management treatments support 5% or greater weight loss among primary care patients and populations with obesity.

## Introduction

Obesity rates continue to rise, with 50% of US adults projected to have obesity by 2030.^1^ The US Food and Drug Administration (FDA) approved semaglutide for weight management in June 2021,^2^ prompting unprecedented demand for and use of injectable antiobesity medications (AOM).^3^ Semaglutide 2.4 mg can support initial and sustained weight loss of 15%,^4,5^ and other AOM are awaiting FDA approval^6^ and undergoing clinical trials.^7,8^ Unfortunately, enthusiasm for these medications is dampened by high cost (>$1000 monthly) and recommendation for lifelong use to prevent weight regain.^9^

Thus, there is an urgent need to understand opportunities to support weight loss among patients with obesity while controlling health care spending. Even modest weight loss of 5% to 10% can help patients prevent and control weight-related conditions, such as type 2 diabetes.^10,11^ Multiple cost-effective weight management treatments (WMT) can support 5% or greater weight loss, including nutrition counseling, medically supervised dietary interventions (eg, very low-calorie meal replacement), oral AOM (phentermine/topiramate, bupropion/naltrexone, or orlistat), and bariatric surgery.^12,13,14,15^ However, less than 5% of eligible individuals receive these WMT,^16,17^ and little is known about their clinical potential to support weight loss among individual patients and populations. To address this gap, we conducted a retrospective cohort study to (1) characterize weight status among patients with established primary care, (2) describe use of WMT, (3) explore associations between WMT and weight trajectories, and (4) estimate the level of WMT engagement necessary to reduce weight at the population level.

## Methods

This is a retrospective cohort study using electronic health record (EHR) data from 1 academic medical center of adult primary care patients between October 1, 2015, and March 23, 2020. We cross-sectionally compared body mass index (BMI; calculated as weight in kilograms divided by height in meters squared), obesity rates, and WMT utilization in 2017 and 2019. Then, we analyzed longitudinal weight trajectories and associations with WMT among patients with obesity (BMI ≥30) at baseline. Specifically, we evaluated weight-change transitions across thresholds of ±5% and ±10% of baseline weight using a novel application of multistate Markov models (MSM). We followed the Strengthening the Reporting of Observational Studies in Epidemiology (STROBE) reporting guidelines.^18^ The study was approved by the University of Michigan Institutional Review Board. Patients did not provide informed consent as this was a secondary analysis of administrative EHR data

### Cohort Selection

We identified patients aged 18 years and older with 2 or more primary care visits 358 or more days apart in the prior 3 years. We truncated eligibility after death, pregnancy, or metastatic cancer diagnosis (eTable 1 in Supplement 1). We excluded patients with less than 2 BMI measurements between 12 and 100 (see the Figure and eFigure 1 and eMethods in Supplement 1).

**Figure.**
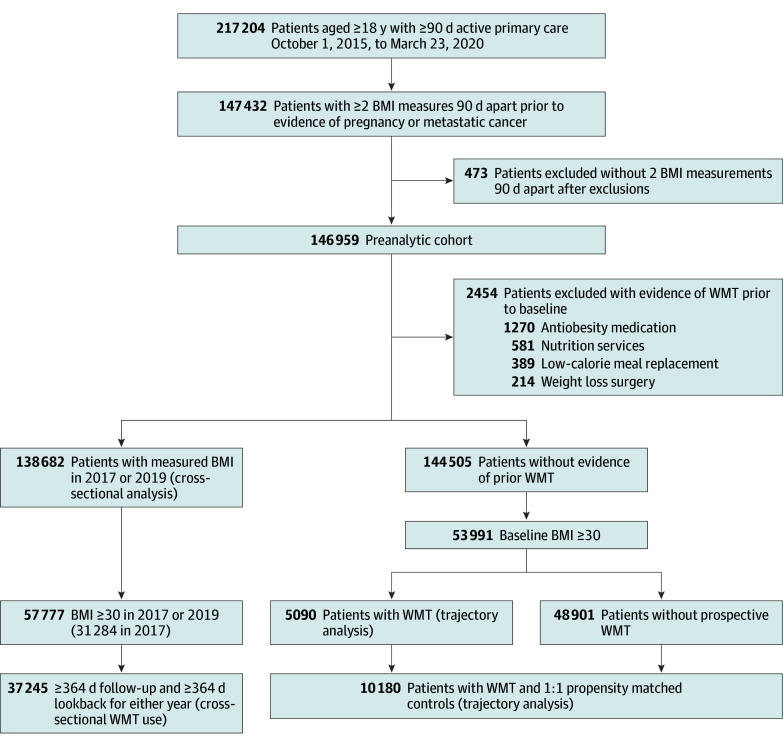
Flow Diagram Showing Cohort Selection Beginning with a cohort of primary care patients, this diagram illustrates inclusion and exclusion criteria to arrive at analytic subgroups. Subgroups used for analyses reported in results are indicated with parentheses. The subgroups used in the primary analyses are labeled cross-sectional analysis and trajectory analysis. BMI indicates body mass index (calculated as weight in kilograms divided by height in meters squared); WMT, weight management treatment.

### Analytic Subsets

We included patients with weight measurements during 2017 and/or 2019 in cross-sectional analyses, analyzing the first weight in each year. We analyzed trajectories for patients with obesity at baseline and without prior known exposure to WMT. We retained patients prospectively exposed to WMT and selected patients without WMT exposure using 1:1 propensity matching. We modeled propensity for exposure to WMT using logistic regression as a function of follow-up time and baseline covariates including age, BMI, sex, and weight-related conditions, including hyperlipidemia, hypertension, nonalcoholic fatty liver disease, obstructive sleep apnea, and type 2 diabetes (T2D). We matched 1:1; adding additional matches would have negligible impact on the precision of our parameter estimates while substantially increasing computational requirements. We matched within 16 strata defined by 4 binary categories: sex (male or female), patient-reported race (White or other [includes Asian, Black, and other/unknown]), patient-reported ethnicity (Hispanic or non-Hispanic), and diagnosis of at least 1 weight-related condition. Propensity matching served to reduce the impact of selection bias and to make MSM computationally tractable. Matching was stratified to ensure balance on the listed factors, each of which could plausibly confound the relationship between WMT exposure and weight status. Race and ethnicity were simplified into majority and nonmajority groups when stratifying to ensure adequate numbers of unexposed patients in all strata

### Outcome, Exposure, and Covariate Definitions

#### Covariates

We used patient-reported sex, race (Asian, Black, White, or other or unknown), and ethnicity (Hispanic, Non-Hispanic, or unknown). Weight-related conditions were identified using diagnosis codes (eTable 2 in Supplement 1).

#### Weight Management Treatments

WMT included nutrition counseling by a registered dietitian (RD), prescriptions for AOM, participation in a very low-calorie meal replacement (MR) program, and bariatric surgery (eTable 3 in Supplement 1). In the MSM, the following are time-varying exposures: nutrition counseling in the prior 90 days; early (months 1-6) or late (months 6-24) participation in the MR program in the prior 90 days; and time elapsed since bariatric surgery divided into 0 to 90 days, 91 to 180 days, 181 to 364 days, and 365 or more days. Using order data, we constructed binary, time-varying exposures to AOM, including orlistat, liraglutide, fixed-combination naltrexone or bupropion, and fixed-combination phentermine or topiramate. To account for off-label prescribing, we also included overlapping exposures to either phentermine and topiramate or bupropion and naltrexone. We identified exposure to glucagon-like peptide-1 receptor agonists (GLP1RA) approved for T2D management during the study period and included these as AOM in the MSM, as these medications support significant weight loss, and some are now approved to treat obesity in patients without T2D (eg, semaglutide).

#### Outcomes and Exposures

Outcomes in cross-sectional analyses were population mean BMI, point prevalence of obesity, and prospective utilization of WMT among patients with obesity along with 364 days of follow-up from index weight measurement. The exposure was year (2017 or 2019). For trajectory analysis, the longitudinal outcome was weight status categorized relative to baseline weight: 10% or greater loss, 5% to 10% loss, within ±5% of baseline, 5% to 10% gain, 10% or greater gain.

### Statistical Analyses

#### Descriptive Statistics

We summarized continuous variables using means and SDs, compared means using linear regression and *t* tests, summarized categorical variables using percentages, compared distributions using χ^2^ tests, and constructed CIs for differences in proportions using large-sample Z tests with continuity correction. *P* values were 2-sided and uncorrected for multiplicity with *P* < .05 considered statistically significant. There were no missing data; however, variable definitions are limited to discrete EHR fields.

#### Cross-Sectional Analysis

We analyzed cross sections using generalized estimating equations with patient clusters and exchangeable correlation. Unadjusted models include only a categorical cross-sectional year. Adjusted models control for age, gender, race, and ethnicity. We used a Gaussian family with identity link for BMI, a binomial family with logit link for obesity prevalence and WMT utilization, and average marginal effects (AMEs) to summarize differences.

#### Trajectory Analysis

We used an MSM to model transitions between weight status categories at observed times (days from baseline).^19^ We used MSM over survival models (eg, proportional hazards) because observations represent patients’ current weight statuses and not times of weight status transitions. Patients transition between adjacent states at rates (log-scale intensities) depending on covariates comprising patient demographics and WMT exposures (eFigure 2 in Supplement 1). We estimated parameters by maximum likelihood, summarized effects with hazard ratios (HR) and 95% CIs, and considered CIs excluding 1 statistically significant.

We also summarized the MSM using 1-year state probabilities, defined as the probability of a weight status after 364 days conditional on a patient’s current status, baseline covariates, and assumed WMT exposures. For each WMT we summarized the 1-year probability of 5% or greater weight loss assuming idealized, uninterrupted exposure for a reference patient (female, age 50, BMI 35, 1 prior-year primary care visit). We collapsed probabilities across states representing weight loss (≥10% or 5%-10% loss), stability (baseline ±5%), or gain (5%-10% or ≥10% gain) and used multivariate-normal simulations to estimate CIs.^20^

We quantified population-level associations of WMT utilization with 5% or greater weight loss after 1 year as the average model-based probability among patients with obesity and 364 or more days of follow-up. We computed the AME of WMT (collectively) as the average difference between this 1-year probability of 5% or greater weight loss and the counterfactual probability assuming no WMT exposure. We contextualized the AME using an observed population attributable fraction, computed by dividing the AME by the observed 1-year probability of 5% or greater weight loss. We estimated AMEs and population attributable fractions for each specific WMT using counterfactuals obtained by zeroing 1 at a time.

To estimate potential population-level outcomes of increased WMT utilization, we investigated counterfactuals representing 2- to 5-fold increases in WMT exposure. We summarized 1-year state probabilities for each counterfactual using the following: the probability of 5% or greater weight loss, the difference between the average probabilities of 5% or greater weight loss and 5% or greater weight gain, and the corresponding population-mean risk ratios. We used SAS version 9.4 (SAS Institute) and R version 4.2.1 (R Project for Statistical Computing) for data management and R version 4.2.2 and version 4.3.0 with packages optmatch,^21^ geepack,^22^ marginaleffects,^23^ and msm^19^ for statistical analyses.^24^ Data were analyzed from October 2021 to October 2023.

## Results

### Primary Care Population

As detailed in the Figure, we identified 146 959 patients meeting inclusion criteria for the preanalytic cohort, with 83 636 female patients (56.9%), 8940 Asian patients (6.1%), 14 560 Black patients (9.9%), 4486 Hispanic patients (3.1%), and 116 664 White patients (79.4%). The mean (SD) age of the cohort was 49.6 (17.7). The mean (SD) baseline BMI was 29.2 (7.2), with 38 182 (26.0%) normal weight (BMI 20-25), 7420 (5.0%) underweight (BMI <20), 45 349 (30.9%) overweight (BMI 25-30), and 55 918 (38.1%) obese (BMI ≥30). We summarize demographics for patients with obesity (BMI ≥30) in Table 1.

**Table 1.** Baseline Characteristics of Primary Care Patients With Body Mass Index Greater Than 30^a^

Characteristic	Patients, No. (%)				
	No exposure to weight management treatments (n = 48 901)	≥1 Nutrition counseling appointment (n = 3364)	≥1 Visit with meal-replacement program (n = 189)	Any antiobesity medication (n = 1428)	Completed bariatric surgery (n = 520)
Age, mean (SD), y	52.1 (15.5)	51.1 (14.6)	45.6 (11.4)	49.1 (12.4)	44.1 (11.2)
Body mass index, mean (SD)^b^	36.0 (5.7)	37.3 (6.3)	39.5 (5.7)	39.9 (7.4)	45.6 (7.2)
Female	26 284 (53.7)	2153 (64.0)	139 (73.5)	932 (65.3)	412 (79.2)
Race					
American Indian and Alaska Native	254 (0.5)	≤11 (≤0.3)	≤11 (≤5.8)	≤11 (≤0.8)	≤11 (≤2.1)
Asian	841 (1.7)	79 (2.3)	<11 (<5.8)	19 (1.3)	≤11 (≤2.1)
Black or African American	6423 (13.1)	591 (17.6)	25 (13.2)	230 (16.1)	100 (19.2)
White	39 407 (80.6)	2521 (74.9)	150 (79.4)	1106 (77.5)	401 (77.1)
Other^c^	1976 (4.0)	162 (4.8)	≤11 (≤5.8)	62 (4.3)	19 (3.7)
Hispanic	1529 (3.1)	141 (4.2)	≤11 (≤5.8)	53 (3.7)	15 (2.0)
Type 2 diabetes	2016 (4.1)	112 (3.3)	≤11 (≤5.8)	146 (10.2)	17 (3.3)
Hyperlipidemia	3712 (7.6)	453 (13.5)	20 (10.6)	220 (15.4)	28 (5.4)
Hypertension	6708 (13.7)	776 (23.1)	27 (14.3)	379 (26.5)	81 (15.6)
Nonalcoholic fatty liver disease	634 (1.3)	56 (1.7)	≤11 (≤5.8)	29 (2.0)	≤11 (≤2.1)
Obstructive sleep apnea	218 (0.4)	22 (0.7)	≤11 (≤5.8)	≤11 (≤0.8)	≤11 (≤2.1)

^a^ Columns represent prospective weight management treatment (WMT) exposures and patients may contribute to more than one exposure column. Patients with prior exposure to WMTs are excluded.

^b^ Body mass index is calculated as weight in kilograms divided by height in meters squared.

^c^ Other includes Native Hawaiian and Other Pacific Islander, other, patient refused, and uknown. Categories are aggregated to limit cell sizes smaller than 11 to preserve patient confidentiality.

### Serial Cross-Sections From 2017 and 2019

Overall, 138 682 patients contributed 214 959 cross-sectional weights with 33 267 patients (24.0%) representing only 2017, 29 138 (21.0%) only 2019, and 76 277 (55.0%) both. We summarize demographics in eTable 4 in Supplement 1.

From 2017 to 2019, average unadjusted BMI increased 0.27 (95% CI, 0.23-0.31) from 29.34 (95% CI, 29.29-29.38) to 29.61 (95% CI, 29.56-29.65); the adjusted increase was similar (AME, 0.21; 95% CI, 0.18-0.25). The unadjusted point prevalence of obesity increased 1.5% (95% CI, 1.1%-2.0%) from 39.2% (95% CI, 38.9%-39.4%) in 2017 to 40.7% (95% CI, 40.4%-41.0%) in 2019; the adjusted estimate was smaller (AME, 0.4%; 95% CI, 0.2%-0.6%). Among 31 284 patients with obesity in 2017, 6665 (25.9%) achieved 5% or greater weight loss at 2 years.

Among 57 777 patients with obesity in either year, we assessed utilization of any WMT among 37 245 (64.5%) who had 364 or more days of follow-up both preceding and following 1 or more weight measurement. These patients had low rates of prospective 1-year WMT utilization in both years (2017, 5.3%; 95% CI, 5.1%-5.6%; 2019, 7.1%; 95% CI, 6.7%-7.4%) with small increases in 2019 (change, 1.7%; 95% CI, 1.3%-2.2%) (Table 2).

**Table 2.** Cross-Sectional Utilization of Weight Management Treatments Among Patients With Obesity in 2017 and 2019^a^

Weight management treatment	Patients, % (95% CI)		Difference, AME (95% CI)^b^ (n = 37 245^c^)
	2017 (n = 30 742)	2019 (n = 17 796)	
Nutrition services	2.8 (2.6 to 3.0)	3.2 (3.0 to 3.5)	0.4 (0.1 to 0.7)
Low-calorie meal replacement	0.6 (0.5 to 0.6)	0.5 (0.4 to 0.7)	−0.0 (−0.2 to 0.1)
Weight-management medications	2.1 (1.9 to 2.2)	3.4 (3.1 to 3.6)	1.3 (0.1 to 1.6)
GLP1RA^d^	2.4 (2.3 to 2.6)	4.2 (3.9 to 4.5)	1.7 (1.4 to 2.1)
Patients without prior bariatric surgery, No.	30 694	17 721	37 1792
Bariatric surgery	0.1 (0.1 to 0.2)	0.3 (0.2 to 0.4)	0.2 (0.1 to 0.2)
Any weight management treatment^e^	5.3 (5.1 to 5.6)	7.1 (6.7 to 7.4)	1.7 (1.3 to 2.2)

Abbreviations: AME, Average Marginal Effect; GLP1RA, glucagon-like peptide-1 receptor agonists.

^a^ Utilization is estimated during the year following a patient’s first weight measurement in each year for patients with a full year of follow-up. We exclude patients with prior bariatric surgery when estimating bariatric surgery utilization.

^b^ Differences are average marginal effect estimates for year from an unadjusted logistic regression model fit using generalized estimating equations clustered on patient.

^c^ Number of unique patients.

^d^ During the study timeframe, GLP1RA were not approved for weight-loss, but are included here separately given their effectiveness for weight-loss.

^e^ Does not include GLP1RA.

### Longitudinal Trajectory Analysis for Patients With Obesity at Baseline

#### Cohort and Time-at-Risk by Exposure

Among 53 991 patients with obesity at baseline and no known prior exposure to WMT, 5090 (9.4%) had prospective exposure to WMT over 16 960 patient-years (PY) of follow-up. Most common was nutrition counseling (3397 patients [6.3%]; 1316 PY). Prescriptions for AOM and GLP1RA (1428 patients [2.6%]; 821 PY) and bariatric surgery (520 patients [1.0%]; 869 PY) were less common. MR program participation was least common (189 patients [0.4%]; 154 PY)^25^ (eTable 5 in Supplement 1).

#### Propensity Matching

We matched 5090 patients with prospective WMT exposure 1:1 to unexposed controls using propensity for WMT exposure (eTable 6 in Supplement 1). All WMT patients were successfully matched; controls contributed 16 589 PY of follow-up. After matching, WMT exposure groups were balanced, with all assessed variables having an absolute standardized mean difference less than 0.1 (eTable 7 in Supplement 1). Together, 10 180 patients (33 549 PY) composed the trajectory analysis cohort.

#### Hazard Ratios for Weight-Loss and Weight-Gain Transitions

We report hazard ratios for WMT exposures in Table 3, and for demographic controls in eTable 8 in Supplement 1. All WMT are associated with increased probability of 5% or greater weight loss from baseline. Additionally, all WMT are associated with increased probability of 5% or greater weight loss when weight is elevated to 5% to 10% above baseline (Table 3). Nutrition counseling (HR, 0.84; 95% CI, 0.74-0.96) and AOM (HR, 0.69; 95% CI, 0.58-0.81) are also associated with decreased probability of weight-gain transitions from baseline. All WMT were associated with decreased probability of weight-gain transition from 5% to 10% loss. Neither MR nor bariatric surgery was significantly associated with weight-gain transitions from baseline, likely due to their weight-loss effectiveness (Table 3 and eTable 5 in Supplement 1).

**Table 3.** Hazard Ratios (HR) for Weight Management Treatments^a^

Transition	HR (95% CI)								
	Nutrition counseling in prior 90 d	Meal replacement program: early active^b^	Meal replacement program: late active	Antiobesity medications	Days since bariatric surgery				
					1-90	91-180	181-365	>365	>90
Weight-loss transitions									
5%-10% loss to >10% loss	1.18 (1.03-1.35)	3.27 (2.63-4.07)	0.49 (0.24-1.00)	0.99 (0.84-1.17)	8.81 (7.84-9.91)	3.79 (2.78-5.17)	1.33 (0.81-2.20)	0.84 (0.56-1.26)	NA
Baseline to 5%-10% loss	1.34 (1.23-1.47)	7.58 (6.31-9.12)	NA	1.56 (1.40-1.74)	27.8 (24.8-31.2)	NA	NA	NA	2.88 (1.90-4.38)
5%-10% gain to baseline	1.14 (1.01-1.29)	5.17 (3.66-7.31)	NA	1.39 (1.20-1.62)	15.8 (12.0-20.7)	NA	NA	NA	NA
>10% gain to 5%-10% gain	1.14 (0.92-1.40)	6.17 (3.84-9.94)	NA	0.99 (0.78-1.26)	11.0 (7.26-16.8)	NA	NA	NA	NA
Weight-gain transitions									
>10% loss to 5%-10% loss	0.93 (0.76-1.15)	0.45 (0.26-0.76)	0.71 (0.51-1.00)	0.79 (0.62-0.99)	0.13 (0.06-0.30)	0.05 (0.02-0.14)	0.08 (0.05-0.13)	0.12 (0.09-0.15)	NA
5%-10% loss to baseline	0.84 (0.74-0.96)	0.60 (0.39-0.93)	0.73 (0.48-1.13)	0.69 (0.58-0.81)	0.15 (0.06-0.38)	0.25 (0.09-0.68)	0.27 (0.11-0.66)	0.36 (0.22-0.59)	NA
Baseline to 5%-10% gain	0.72 (0.64-0.82)	1.07 (0.60-1.91)	NA	0.80 (0.67-0.95)	0.72 (0.25-2.04)	NA	NA	NA	0.50 (0.19-1.34)
5%-10% gain to >10% gain	0.93 (0.77-1.13)	0.79 (0.22-2.86)	NA	0.85 (0.65-1.11)	0.38 (0.03-5.71)	NA	NA	NA	NA

Abbreviation: NA, not applicable.

^a^ Hazard ratios for weight management treatments from a multistate Markov model assessing patients’ longitudinal weight status.

^b^ Patients in the meal-replacement program were considered active if they had a visit in the prior 90 days. The early phase consisted of the first 6 months of the program and the late phase of months 7 to 24.

#### One-Year Probabilities of 5% or Greater and 10% or Greater Weight Loss

For reference control without WMT exposure, the expected 1-year probabily of 5% or greater weight loss was 15.6% (95% CI, 15.3%-16.5%; ≥10% weight loss, 5.0%; 95% CI, 4.8%-5.4%). Relative to control, 5% or greater and 10% or greater weight loss was more likely for patients with year-long exposure to any WMT (Table 4). The 1-year probabilities of 5% or greater weight loss were 23.1% for nutrition counseling (95% CI, 21.3%-25.1%; ≥10% weight loss, 8.4%; 95% CI, 7.3%-9.5%), 54.6% for MR (95% CI, 46.5%-61.3%; ≥10% weight loss, 29.6%; 95% CI, 23.3%-36.6%), 27.8% for AOM (95% CI, 25.0%-30.5%; ≥10% weight loss, 9.3%; 95% CI, 7.9%-10.8%), and 93.0% for bariatric surgery (95% CI, 89.7%-95.0%; ≥10% weight loss, 83.5%; 95% CI, 79.0%-87.0%).

**Table 4.** Expected Probability of 5% or Greater and 10% or Greater Weight Loss at 1 Year^a^

Weight status	1-y probability or difference in probability, % (95% CI)								
	Reference^b^	Nutrition services		Low-calorie meal replacement		Weight management medications		Bariatric surgery	
	1-y	1-y	Change^c^	1-y	Change^c^	1-y	Change^c^	1-y	Change^c^
≥10% Weight loss	5.0 (4.8 to 5.4)	8.4 (7.3 to 9.5)	3.4 (2.3 to 4.4)	29.6 (23.3 to 36.6)	24.6 (18.3 to 31.5)	9.3 (7.9 to 10.8)	4.2 (2.8 to 5.6)	83.5 (79.0 to 87.0)	78.5 (74.1 to 81.9)
≥5% Weight loss^d^	15.6 (15.3 to 16.5)	23.1 (21.3 to 25.1)	7.5 (5.7 to 9.4)	54.6 (46.5 to 61.3)	38.9 (31.0 to 45.6)	27.8 (25.0 to 30.5)	12.2 (9.5 to 14.8)	93.0 (89.7 to 95.0)	77.3 (74.1 to 79.4)
Weight stable^e^	67.7 (66.9 to 68.5)	65.2 (63.2 to 67.4)	−1.4 (−3.6 to 0.6)	38.7 (32.6 to 45.7)	−27.9 (−34.0 to −21.1)	61.4 (58.6 to 64.0)	−5.3 (−8.0 to −2.6)	6.3 (4.2 to 9.2)	−60.4 (−62.5 to −57.3)
≥5% Weight gain^f^	16.4 (15.8 to 17.0)	11.6 (10.3 to 13.1)	−6.1 (−7.5 to −4.6)	6.7 (5.5 to 8.4)	−0.110 (−12.1 to −9.3)	10.8 (9.1 to 12.8)	−6.9 (−8.6 to −5.0)	0.8 (0.4 to 1.9)	−16.9 (−17.7 to −15.6)
≥10% Weight gain	4.8 (4.4 to 5.1)	2.9 (2.4 to 3.5)	−1.9 (−2.4 to −1.2)	1.3 (1.0 to 2.0)	−3.4 (−3.8 to −2.8)	2.7 (2.1 to 5.1)	−2.0 (−2.7 to −1.2)	0.2 (0.1 to 0.5)	−4.6 (−4.9 to −4.2)

^a^ In this table we report the probability of 5% or greater and 10% or greater weight loss, weight stable, or weight gain from baseline after 1 year. Probabilities are estimated from a multistate trajectory model. These are probabilities at the individual patient level under an idealized assumption of continuous treatment exposure with nontreatment covariates set to reference levels.

^b^ Age 50 years, female, body mass index 35 (calculated as weight in kilograms divided by height in meters squared), 1 primary care visit in prior year.

^c^ Difference from the reference control.

^d^ Inclusive of 10% or greater weight loss.

^e^ Baseline weight ±5%.

^f^ Inclusive of 10% or greater weight gain.

#### Population-Level Associations and Attributable Fractions

While these probabilities demonstrate associations of WMT with weight loss for individual patients, low utilization may limit population-level reach. To characterize population-level associations between 5% or greater weight loss and WMT during the study period, we compare average model-based 1-year probabilities of 5% or greater weight loss under observed WMT use during the first year of follow-up with a counterfactual probability assuming no WMT treatment among 47 280 patients followed up for 364 or more days. Under observed WMT use, the average 1-year probability of 5% or greater weight loss is 17.6% (95% CI, 17.1%-18.0%) compared with 17.1% (95% CI, 16.6%-17.6%) assuming no WMT. This is a difference of 0.50% (95% CI, 0.48%-0.52%) and a population attributable fraction of 0.50 ÷ 17.6 = 2.8% (95% CI, 2.6%-3.0%). See eTable 9 in Supplement 1 for specific WMT.

#### Counterfactual Probabilities of 5% or Greater Weight Loss for Hypothetical Increases in WMT Utilization

To understand how increased WMT utilization might be associated with population-level weight trajectories, we estimated counterfactuals with WMT use increased 2- to 5-fold. Each additional 1-fold increase in WMT use increased the 1-year expected proportion of patients with 5% or greater weight loss by 0.5%. Estimated proportions for 2- to 5-fold increases were, respectively, 18.5% (95% CI, 18.1% to 19.0%), 19.0% (95% CI, 18.6% to 19.5%), 19.5% (95% CI, 19.1% to 20.0%), and 20.0% (95% CI, 19.6% to 20.5%). One important threshold for population-level weight management is the probability of 5% or greater weight loss exceeding that of 5% or greater weight gain. This is a tipping point where, all else equal, the proportion of individuals in weight-loss states would exceed the proportion in weight-gain states over time. Under the status quo, the expected proportions of 5% or greater weight gain and 5% or greater weight loss are not statistically different (loss less gain difference, −0.23%; 95% CI, −0.95% to 0.47%; relative risk, 0.99; 95% CI, 0.95 to 1.03). However, a 2-fold increase in WMT utilization would suffice to make the 1-year probability of 5% or greater weight loss significantly higher than for 5% or greater weight gain (difference, 1.11%; 95% CI, 0.40% to 1.79%; relative risk, 1.06; 95% CI, 1.02 to 1.11). For 3- to 5-fold increases the differences are, respectively, 1.78% (95% CI, 1.07% to 2.47%), 2.45% (95% CI, 1.74% to 3.13%), and 3.10% (95% CI, 2.37% to 3.78%) and the relative risks are 1.10 (95% CI, 1.06 to 1.15), 1.14 (95% CI, 1.10 to 1.19), and 1.18 (95% CI, 1.14 to 1.23).

## Discussion

To our knowledge, this is the first longitudinal cohort study using EHR data to examine both WMT utilization and population-level associations between WMT use and weight loss among primary care patients with obesity. Moreover, this article makes a novel contribution by projecting to what extent WMT use would need to expand to have more patients achieving 5% or greater weight loss than achieving 5% or greater weight gain.

From 2017 to 2019, the prevalence of obesity among 138 682 primary care patients increased by 1.5% (39.2% vs 40.7%). Among 31 284 patients with obesity in 2017 and follow-up weight data in 2019, 25.9% achieved 5% or greater weight loss. Prior work has shown that between 10% and 27% of patients with obesity achieve 5% or greater weight loss over 6 months to 5 years,^26,27,28,29^ with a greater annual probability of achieving 5% or greater weight loss observed among individuals with a higher baseline BMI (≥35).^27,28^

Our findings provide novel insight into weight management in primary care settings by characterizing the use and effectiveness of WMT, including nutrition counseling with a dietitian, very low-calorie MR, AOM, and bariatric surgery. The overall rate of WMT utilization increased during the 2-year observation period but remained low at 7.1% in 2019. Without WMT exposure, the 1-year probability of achieving 5% or greater weight loss for a reference control was 15.6%. In our study, the annual probability of 5% or greater weight loss was estimated to increase with year-long exposure to any WMT, ranging from 23.1% for nutrition counseling to 93.0% for bariatric surgery. Additionally, WMT were associated with reduced probability of weight gain. These estimates reflect idealized circumstances, as most patients in real-world settings do not remain continuously engaged in WMT for 1 year.^30^ Efforts to help patients with obesity achieve and maintain 5% or greater weight loss should focus on increasing initial uptake and sustaining engagement in WMT.

Previously, little was known about the extent to which WMT support population-level weight loss. Among our cohort of patients with obesity, the average 1-year probability of achieving 5% or greater weight loss was 17.6%, with less than 1% associated with exposure to the WMT examined. This may reflect weight-loss efforts outside the health system, as approximately 60% of US adults with obesity attempted to lose weight in the prior year.^31^ Despite the low fraction of 5% or greater weight loss associated with WMT, we found a 2-fold proportional increase in WMT use would be sufficient to make the probability of 5% or greater weight loss more likely than 5% or greater weight gain. While we included GLP1RA for T2D, including semaglutide 1.0 mg, in our analyses, the study period predated the FDA-approval of semaglutide 2.4 mg for weight management. Future work should explore the potential for semaglutide 2.4 mg and other medications with substantial weight loss effectiveness to reduce weight at the population level. Additionally, given their potential weight-loss effectiveness and lower cost, future work should explore strategies to enhance patient-centered use of all WMT.

### Limitations

This study has several limitations. First, it was conducted using EHR data from a single academic health system. Second, weight data and WMT exposures were extracted from the EHR and may be subject to measurement error, and we also lack information on WMT adherence. Third, we only capture WMT exposures delivered through the health system and not those delivered by outside practitioners or programs, or self-initiated by patients. Fourth, because this was a retrospective, observational study, our estimates of WMT effectiveness may be biased by treatment selection effects, such as patients’ preferences, clinicians’ practice patterns, and insurers’ coverage for treatment options. Fifth, our study period occurred before FDA approval of semaglutide for weight management, and thus our findings may understate current use and effectiveness of AOM.

## Conclusions

In this cohort study of primary care patients with obesity, we demonstrated meaningful associations between weight management treatments (WMT) and 5% or greater weight loss for individuals. Yet, low rates of WMT utilization hindered population-level benefit. Health systems and insurers should consider novel strategies to enhance preference-sensitive use of WMT to optimize achievement of 5% or greater weight loss among individuals and populations with obesity.

## Notes

Supplement 1. **eTable 1. **Procedure Codes Used for Pregnancy Exclusion **eFigure 1. **Schematic Illustrating Time Periods Related to Cohort Selection **eMethods. **Primary Care Cohort Selection **eTable 2. ***International Statistical Classification of Diseases and Related Health Problems, Tenth Revision* and *International Classification of Diseases, Ninth Revision* Diagnosis Codes for Weight-Related Conditions **eTable 3. **Procedure Codes Used to Identify Weight Loss Surgery **eFigure 2. **Illustration of Weight-Loss Status States Used in the Multistate Markov Model **eTable 4. **Demographics for Serial Cross-Sections Analysis **eTable 5. **Unique Patients and Time at Risk by Exposure and State **eTable 6. **Odds Ratios From Propensity Score Model for Probability of Prospective Weight Management Treatment Exposure **eTable 7. **Balance Assessment for Propensity Matching **eTable 8.** Hazard Ratios for Control Covariates **eTable 9. **Marginal Effects and Observed Population Attributable Fractions for Individual Weight Management Treatments

Supplement 2.
Data Sharing Statement
