# Supplementary material for: Weight Loss Treatment and Longitudinal Weight Change Among Primary Care Patients With Obesity
Source: JAMA Netw Open. 2024 Feb 15;7(2):e2356183. doi: 10.1001/jamanetworkopen.2023.56183 (PMC10870179; doi:10.1001/jamanetworkopen.2023.56183)
Supplement: Supplement 1. — eTable 1. Procedure Codes Used for Pregnancy Exclusion eFigure 1. Schematic Illustrating Time Periods Related to Cohort Selection eMethods. Primary Care Cohort Selection eTable 2. International Statistical Classification of Diseases and Related Health Problems, Tenth Revision and International Classification of Diseases, Ninth Revision Diagnosis Codes for Weight-Related Conditions eTable 3. Procedure Codes Used to Identify Weight Loss Surgery eFigure 2. Illustration of Weight-Loss Status States Used in the Multistate Markov Model eTable 4. Demographics for Serial Cross-Sections Analysis eTable 5. Unique Patients and Time at Risk by Exposure and State eTable 6. Odds Ratios From Propensity Score Model for Probability of Prospective Weight Management Treatment Exposure eTable 7. Balance Assessment for Propensity Matching eTable 8. Hazard Ratios for Control Covariates eTable 9. Marginal Effects and Observed Population Attributable Fractions for Individual Weight Management Treatments [file jamanetwopen-e2356183-s001.pdf]

Supplemental Online Content

Henderson J, Ehlers AP, Lee JM, et al. Weight loss treatment and longitudinal weight change among primary care patients with obesity. *JAMA Netw Open*. 2024;7(2):e2356183. doi:10.1001/jamanetworkopen.2023.56183

eTable 1. Procedure Codes Used for Pregnancy Exclusion
eFigure 1. Schematic Illustrating Time Periods Related to Cohort Selection
eMethods. Primary Care Cohort Selection
eTable 2. *International Statistical Classification of Diseases and Related Health Problems, Tenth Revision* and *International Classification of Diseases, Ninth Revision* Diagnosis Codes for Weight-Related Conditions
eTable 3. Procedure Codes Used to Identify Weight Loss Surgery
eFigure 2. Illustration of Weight-Loss Status States Used in the Multistate Markov Model
eTable 4. Demographics for Serial Cross-Sections Analysis
eTable 5. Unique Patients and Time at Risk by Exposure and State
eTable 6. Odds Ratios From Propensity Score Model for Probability of Prospective Weight Management Treatment Exposure
eTable 7. Balance Assessment for Propensity Matching
eTable 8. Hazard Ratios for Control Covariates
eTable 9. Marginal Effects and Observed Population Attributable Fractions for Individual Weight Management Treatments

This supplemental material has been provided by the authors to give readers additional information about their work.

© 2024 Henderson J et al. *JAMA Network Open.*

eTable 1. Procedure Codes Used for Pregnancy Exclusion

Exclusion	Code Set	Codes
Pregnancy		
Antepartum visits	CPT4	0500F, 0501F, 0502F, 0503F
Postpartum Visits	CPT4	0503F, 59430
Delivery	CPT4	594xx, 595xx, 596xx, 598xx

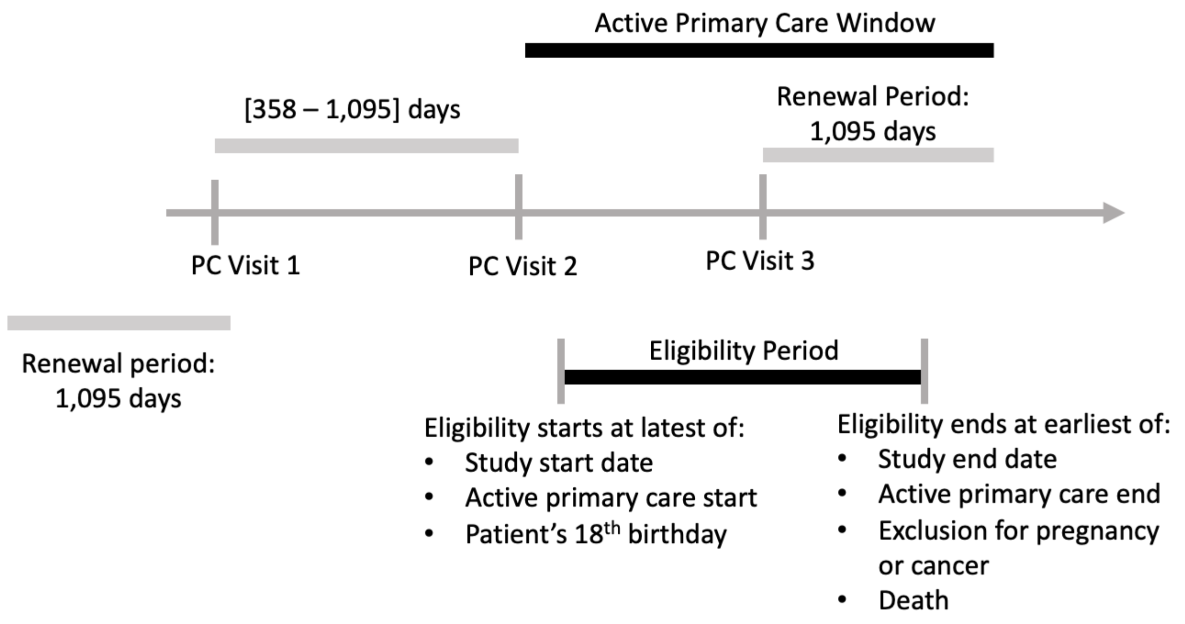

eFigure 1. Schematic Illustrating Time Periods Related to Cohort Selection

eMethods. Primary Care Cohort Selection

Active Primary Care

In this section we provide additional detail on how we identified periods of active primary care using data on primary care visits from the EHR. First, we identified all primary care visits between October 1, 2012, and March 23, 2020. We defined primary care visits as completed visits with a primary care provider, based on the provider's designated provider type.

© 2024 Henderson J et al. *JAMA Network Open.*

Next, we identified all instances in which a patient had two primary care visits separated by [358 – 1,095] days. For each such instance, we defined the day of the second primary care visit as an index date. Then, using a renewal period of 1,095 days (~3 years), we identified periods of active primary care ending 1,095 days after the final primary care visit within the renewal period. Therefore, patients with active primary care all had at least two initial primary care visits separated by $\geq$ 358 days and had at least one primary care visit in the prior 3 years (1,095 days). See eFigure 1.

Cohort Eligibility

Patients were eligible for study inclusion during a period of active primary care. The start of each patient's eligibility period was the latest date among the beginning of the study period (October 1, 2015), the start of their active primary care window, or their 18th birthday. Likewise, the end of each patient's eligibility period was the earliest of the end of their active primary care window, the study period end date (March 23, 2020), or death. We initially included patients with $\geq$ 90 days of active primary care after these truncations and used the longest such window from patients with more than qualifying instance.

Exclusion Criteria

After identifying windows of active primary care as just described we excluded patients without 2 valid body mass index measurements (BMI) $\geq$ 90 days apart, with the valid range considered 12-100 kg/m^2. Next, we additional excluded patients with evidence of pregnancy or metastatic cancer by truncating their period of eligibility (active primary care window) using the following rules. We excluded potentially pregnant patients and patients with metastatic cancer because we considered the potential weight changes associated with these conditions outside the scope of this study. For pregnancy, we truncated eligibility at the earlier of 10 weeks before an

© 2024 Henderson J et al. *JAMA Network Open.*

antepartum visit, 40 weeks before a delivery, or 44 weeks before a postpartum visit. For cancer, we truncated eligibility 6 months prior to the first evidence. After truncation, we excluded patients without at least 2 valid BMI measurements $\geq$ 90 days apart. We identified pregnancy related visits using Current Procedural Terminology (CPT4) codes; see eTable 1. We identified metastatic cancer using a pre-built flag based on the Elixhauser comorbidity index in our research data warehouse.

Weight Management Treatment Definitions

In this section we elaborate on the weight management treatment definitions.

Nutrition Counseling by a Registered Dietitian

We defined exposure to nutrition counseling by a Registered Dietitian (RD) using department and clinic names from visit data. In cross-sectional analyses we counted the number of unique days with visits during the follow-up periods. In the trajectory analysis, the time-varying nutrition counseling exposure is a flag representing whether a patient had one or more nutrition counseling visits in the prior 90 days.

Anti-Obesity Medications

We used provider orders to identify patients with exposure to AOMs, including orlistat, liraglutide, fixed-combination naltrexone/bupropion, and fixed-combination phentermine/topiramate. To account for off-label prescribing of brand name medications, we also included any overlapping exposures to either phentermine and topiramate or bupropion and naltrexone. We identified prescriptions from orders data using both generic and name brand drug names. The prescribed quantity of AOMs, including any refills, and the defined daily dose were used to construct an estimate of the number of days ordered. We assumed patients began taking the medication the day it was ordered and defined a patient as exposed to a medication for the

© 2024 Henderson J et al. *JAMA Network Open.*

estimated number of active prescription days. Patients were assumed to have continuous exposure to a medication if there were fewer than 14 days between the expiration of one order and the start of the next.

Very low-calorie meal replacement

We identified patients participating in a very low-calorie meal replacement (MR) program using visits at the program location with program providers. This is a 24-month program, consisting of an initial and interval visits with a program physician, regular meetings with a program RD, physical activity recommendations, an initial intensive energy restriction phase of 800-900 kcal/d in the form of MR products (shakes +/- soups) for 3 months, and a gradual transition to a low calorie conventional food based diet.(cite) We considered patients active participants starting from their first visit with an RD at the program site, provided they had a visit with a program physician in the prior 90 days. We continued to consider patients active for any period over the subsequent two years in which they had a visit with any program provider (either an RD or physician) in the prior 90 days. Exposure to the MR program was divided into an "early" phase consisting of the first 6 months of engagement and a "late" phase encompassing months 6-24.

Bariatric Surgery

We identified bariatric surgery using procedure codes as detailed in eTable 4. When treated as an outcome in the cross-sectional analysis, we excluded patients with prior bariatric surgery. In the trajectory analysis, the time-varying bariatric surgery exposure was divided into indicators representing distinct periods after surgery: [0-90) days, [90-180) days, [180-365) days and ≥ 365 days.

© 2024 Henderson J et al. *JAMA Network Open.*

eTable 2. *International Statistical Classification of Diseases and Related Health Problems, Tenth Revision* and *International Classification of Diseases, Ninth Revision* Diagnosis Codes for Weight-Related Conditions

**Condition**	**ICD-10**	**ICD-9**
Non-alcoholic fatty liver disease	K7581, K760, K740, K769	571.5, 571.8, 571.9
Obstructive sleep apnea	G4730, G4733, G4739	780.53, 780.57
Hyperlipidemia	E78.0, E78.00, E78.01, E78.1, E78.2, E78.2, E78.4, E78.41, E78.49, E78.5	272.0, 272.1, 272.2, 272.3, 272.4
Hypertension	H35031, H35.032, H35.033, H35.039, I10, I11.0, I11.9, I12.0, I12.9, 113.0, I13.10, I13.11, I13.2, I14.0, I15.1, I15.2, I15.8, I15.9, I67.4, N26.2	362.11, 401.0, 401.1, 401.9, 402.01, 402.11, 402.90, 402.91, 403.00, 403.01, 403.10, 403.11, 403.90, 403.91, 404.00, 404.01, 404.02, 404.03, 404.10, 404.11, 404.12, 404.13, 404.90, 404.91, 404.92, 404.93, 405.01, 405.09, 405.11, 405.19, 405.91, 405.99, 437.2
Type 2 Diabetes Mellitus	E11 (all codes)	250.00, 250.02, 250.10, 250.12, 250.20, 250.22, 250.30, 250.32, 250.40, 250.42, 250.50, 250.52, 250.60, 250.62, 250.70, 250.72, 250.80, 250.82, 250.90, 250.92

eTable 3. Procedure Codes Used to Identify Weight Loss Surgery

**Procedure**	**Code Set**	**Codes**
Weight loss surgery	CPT4	43644, 43645, 43770, 43775, 43842, 43843, 43846, 43847, 43845, 43848

© 2024 Henderson J et al. *JAMA Network Open.*

Multistate Markov Models

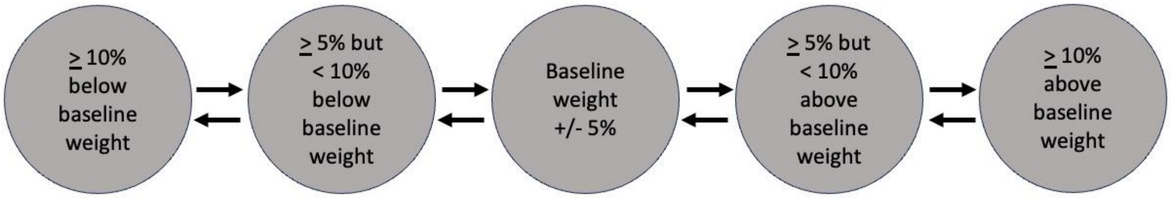

eFigure 2. Illustration of Weight-Loss Status States Used in the Multistate Markov Model (MSM)
Circles represent weight-loss statuses observed within the electronic health record computed as percentages of each patient's baseline weight. Arrows represent transitions or changes in status which (here) are only allowed between adjacent states. The Markov model estimates the rates of transitions which depend on covariates which include time-varying exposures to weight loss treatment and baseline demographics. Conditional on being in one of these 5 states at a given point in time, the probabilities of being in any of the 5 states at a specific time in the future is computed by integrating over all possible transition combinations during the intervening time. In practice this is expressed through a mathematical concept known as matrix exponentiation and through algorithms for approximating these.

The longitudinal analysis of patients' weight status trajectories in this paper uses a Multistate Markov Model (MSM). Here "multistate" refers to one of 5 "states" representing a patient's weight status relative to baseline. These 5 states are illustrated in eFigure2. Each time a patient's weight is measured by the health system during their follow-up period we observe their weight status – or state – at that point in time, by comparing the weight measurement to their baseline weight.

In a traditional time-to-event or survival analysis the dependent variable is the timing of *transitions* from one state to another, e.g. the timing of a patient achieving 5% weight loss and transitioning from the "baseline +/- 5%" state to "≥ 5% but < 10% below baseline state". However, such models generally assume we observe the timing of the transition directly rather than observing their weight status or state at arbitrary times as we do here. Data like ours are known as *current-status* data and can be modeled using MSMs.

© 2024 Henderson J et al. *JAMA Network Open.*

The “Markov” in Multistate Markov model refers to the Markov property – an assumption that the probability of each future state depends only on the current state and exposures and not on prior states. The instantaneous probabilities of transitions between adjacent states – or hazards – are modeled on the log-scale. The log hazards – or intensities – depend linearly on baseline covariates and time-varying exposures. The coefficients expressing this dependence are estimated using maximum likelihood. The likelihood is calculated from the probability of a patient being in observed states at observed times. Because of the Markov property, this likelihood is a product of successive pairs of observed states for each patient. The probability that a patient in state x at time t is in state y at time s, is computed by integrating over all combinations of transitions leading from state x to state y in time $s - t$. Although there are infinitely many combinations of transitions, the integral can be expressed as a matrix exponential for which there exist approximation algorithms.

© 2024 Henderson J et al. *JAMA Network Open.*

eTable 4. Demographics for Serial Cross-Sections Analysis

Variable	Both, N (%)	2017 only, N (%)	2019 only, N (%)	Change (95% CI); p-value (2019 only less 2017 only)
Total, N	76,277	33,267	29,138	-
Age, mean (IQR)	53.1[1] (42.4-64.9)	48.8 (32.2-63.2)	48.5 (32.7-62.9)	-0.26 (-0.55 to 0.04); p = 0.09
Gender				p < 0.001
Female[2]	43,239 (56.7)	19,382 (58.3)	16,188 (55.6)	-2.7 (-3.5 to -1.9)
Male	33,038 (43.3)	13,885 (41.7)	12,950 (44.4)	2.7 (1.9-3.5)
Race				p < 0.001
American Indian or Alaska Native	308 (0.4)	108 (0.3)	137 (0.5)	0.1 (0.0-0.2)
Asian	4,585 (6.0)	2,016 (6.1)	1,818 (6.2)	0.2 (-0.2 to 0.6)
Black or African American	7,584 (10.3)	2,943 (8.8)	2,943 (10.1)	1.3 (0.8-1.7)
Native Hawaiian or Other Pacific Islander	57 (0.1)	34 (0.1)	14 (0.0)	-0.1 (-0.1 to -0.0)
White or Caucasian	60,432 (79.2)	26,807 (80.6)	22,901 (78.6)	-2.0 (-2.6 to -1.4)
Other[3]	3,041 (4.0)	1,359 (4.1)	1,325 (4.5)	0.5 (0.1-0.8)
Ethnicity				p < 0.001
Hispanic	2,156 (2.8)	981 (2.9)	1,088 (3.7)	0.8 (0.5-1.1)
Non-Hispanic	70,664 (92.6)	30,818 (92.6)	27,202 (93.4)	0.7 (0.3-1.1)
Unknown	3,457 (4.5)	1,468 (4.4)	848 (2.9)	-1.5 (-1.8 to -1.2)
Type-2 Diabetes	1,608 (2.1)	485 (1.5)	1,824 (6.3)	4.8 (4.5-5.1); p < 0.001
Hyperlipidemia	7,080 (9.3)	1,624 (4.9)	441 (1.5)	-3.4 (-3.6 to -3.1); p < 0.001
Hypertension	11,265 (14.8)	2,896 (8.7)	645 (2.2)	-6.5 (-6.8 to -6.1)
Non-alcoholic fatty liver disease	937 (1.2)	354 (1.1)	40 (0.1)	-0.9 (-1.0 to -0.8); p < 0.001
Obstructive sleep apnea	294 (0.4)	85 (0.3)	≤ 11 (< 0.1)	-0.2 (-0.3 to -0.2); p < 0.001

1. Age are at time of BMI measurement, with age at the 2017 measurement used for 'Both'.
2. ≤11 individuals with undefined gender grouped with Female.
3. Includes Other, Patient Refused, and Unknown.

© 2024 Henderson J et al. *JAMA Network Open.*

eTable 5. Unique Patients and Time at Risk by Exposure and State

	Weight Category State, n (person years at risk)					
Exposure	≥ 10% loss	5-10% loss	Baseline	5-10% gain	≥ 10% gain	Total
Total	2,800 (2,855)	4,590 (3,331)	10,180 (22,646)	3,776 (3,138)	1,663 (1,580)	10,180 (33,549)
Weight Status Measures, n	29,182	30,613	174,367	28,311	15,952	278,425
Controls						
Male	916 (851)	1,585 (1,164)	3,536 (8,263)	1,160 (960)	453 (430)	3,536 (11,700)
1 PCP visit in past 365 days	1,427 (603)	2,190 (811)	7,684 (6,018)	2,115 (817)	918 (387)	8,486 (8,660)
2 PCP visits in past 365 days	2,401 (2,051)	3,871 (2,252)	9,437 (14,640)	3,118 (2,013)	1,345 (1,038)	9,697 (22,054)
Weight Management Treatments						
≥1 Nutrition Counseling Appointment in prior 90 days	252 (81)	572 (139)	2,702 (895)	555 (127)	242 (63)	3,397 (1,316)
Low calorie meal replacement - Early Active	82 (27)	104 (19)	164 (34)	40 (4)	24 (3)	189 (88)
Low calorie meal replacement - Late Active	65 (37)	47 (12)	45 (14)	≤ 11 (< 1)	≤ 11 (< 1)	111 (66)
Weight management medications	240 (116)	359 (116)	1,048 (450)	300 (81)	166 (55)	1,428 (821)
Days Since Bariatric Surgery						
1-90	341 (45)	330 (34)	354 (33)	57 (3)	29 (3)	520 (119)
91-180	372 (82)	101 (17)	50 (8)	-	-	446 (107)
181-365	403 (171)	67 (12)	25 (5)	-	-	428 (189)
> 365 (weight loss states)	321 (410)	53 (23)	-	-	-	330 (432)
> 90 (baseline)	-	-	62 (22)	-	-	62 (22)

© 2024 Henderson J et al. *JAMA Network Open.*

eTable 6. Odds Ratios From Propensity Score Model for Probability of Prospective Weight Management Treatment Exposure

Characteristic	Odds Ratio (95% CI)
Intercept, probability	0.01(0.01-0.02)
Age (decades)[1]	0.86 (0.84-0.88)
Baseline BMI (kg/m^2)	1.06 (1.05-1.06)
Male	0.67 (0.63-0.72)
Race[2]	
Asian	1.25 (1.01-1.56)
Black or African American	1.09 (1.01-1.19)
Other[3]	1.09 (0.91-1.31)
Unknown or Patient Refused	0.92 (0.67-1.01)
Weight-related conditions	
Hyperlipidemia	1.46 (1.32-1.61)
Hypertension	1.29 (1.19-1.41)
Non-alcoholic fatty liver disease	1.07 (0.85-1.35)
Obstructive Sleep Apnea	0.70 (0.46-1.05)
Type 2 Diabetes Mellitus	2.11 (1.82-2.44)
Follow Up[3] (years)	1.70 (1.65-1.75)

BMI = Body Mass Index, CI = Confidence Interval

[1] Age is centered at the mean of 51.9 years and scale by 10 to represent decades.

[2] Reference category is White or Caucasian.

[3] Follow up is centered at a mean of 2.68 years.

[4] American Indian or Alaska Native, Native Hawaiian or Other Pacific Islander, Other, Multiple

© 2024 Henderson J et al. *JAMA Network Open.*

eTable 7. Balance Assessment for Propensity Matching

In this table we assess covariate balance after propensity matching patients with weight management treatment exposures to those without. Matching was 1:1 and done within 16 strata defined by 4 binary variables: gender, race (White vs non-white), ethnicity (Hispanic vs non-Hispanic/unknown), and presence of any obesity related condition (hyperlipemia, hypertension, non-alcoholic fatty liver disease, obstructive sleep apnea or type 2 diabetes). By design, the cohorts were perfectly balanced by gender, White race, and Hispanic ethnicity. A Kolmogorov-Smirnov test failed to identify differences in the distributions of the linear predictors for the propensity (D = 0.002, p = 1.00).

**Characteristic**	**WMT Exposed, N (%)**	**No WMT Exposure, N (%)**	**Standardized Mean Difference**
Baseline Age, mean (sd)	49.9 (13.9)	48.6 (15.0)	0.092
Baseline BMI, mean (sd)	38.7 (7.1)	38.9 (7.6)	-0.029
Days at risk, mean (sd)	1213.7 (399.2)	1187.1 (399.5)	0.066
Ethnicity			
Non-Hispanic	4,705 (92.4)	4,754 (93.4)	-0.038
Unknown	185 (3.6)	136 (2.7)	0.055
Self-reported Race			
Black or African American	878 (17.2)	863 (17.0)	0.008
Asian	98 (1.9)	91 (1.8)	0.010
Other	209 (4.1)	226 (4.4)	-0.017
Unknown	46 (0.9)	51 (1.0)	-0.010
Obesity-Related Conditions			
Hyperlipidemia	674 (13.2)	656 (12.9)	0.010
Hypertension	1,160 (22.8)	1,172 (23.0)	-0.006
Non-alcoholic fatty liver disease	88 (1.7)	112 (2.2)	-0.034
Obstructive Sleep Apnea	28 (0.6)	23 (0.5)	0.014
Type 2 Diabetes	260 (5.1)	306 (6.0)	-0.039

WMT = Weight Management Treatment

© 2024 Henderson J et al. *JAMA Network Open.*

eTable 8. Hazard Ratios for Control Covariates
This table contains hazard ratios and 95% confidence intervals for control covariates from the multistate trajectory model described in the main paper. Hazard ratios for primary weight management treatment exposures are given in Table 2 of the main manuscript.

**Transition**	**Male**	**Age (1 SD)**	**Baseline BMI (per 5 kg/m^2)**	**365-day PCP visits (0-2)**
Weight Loss Transitions				
5-10% loss to > 10% loss	1.07 (1.00-1.15)	0.92 (0.89, 0.95)	1.07 (1.04, 1.09)	1.27 (1.18-1.35)
baseline to 5-10% loss	1.07 (1.02-1.12)	0.98 (0.96, 1.01)	1.08 (1.07, 1.10)	1.25 (1.20-1.31)
5-10% gain to baseline	1.09 (1.03-1.16)	1.05 (1.01, 1.08)	1.02 (1.00, 1.04)	1.24 (1.18-1.31)
> 10% gain to 5-10% gain	1.03 (0.93-1.14)	1.29 (1.22, 1.35)	1.06 (1.02, 1.09)	1.24 (1.14-1.36)
Weight Gain Transitions				
> 10% loss to 5-10% loss	1.27 (1.17-1.38)	1.01 (0.96-1.05)	0.96 (0.93-0.98)	1.19 (1.09-1.28)
5-10% loss to baseline	1.13 (1.07-1.20)	0.88 (0.86-0.91)	0.99 (0.97-1.01)	1.28 (1.21-1.35)
baseline to 5-10% gain	0.85 (0.81-0.90)	0.73 (0.71-0.75)	0.97 (0.96-0.99)	1.18 (1.13-1.24)
5-10% gain to > 10% gain	0.91 (0.83-0.99)	0.81 (0.78-0.85)	0.97 (0.94-0.99)	1.36 (1.26-1.46)

© 2024 Henderson J et al. *JAMA Network Open.*

eTable 9. Marginal Effects and Observed Population Attributable Fractions for Individual Weight Management Treatments (WMT)

We estimated the marginal effect of each class of WMT as utilized during the study period by comparing to a counterfactual assuming no patients receive that class of WMT, while holding other WMTs and control covariates fixed. Estimate are averaged over 47,280 patients with obesity and a full year of follow up from baseline. The observed population attributable fraction is the marginal effect over the reference probability from WMT use as observed.

WMT Counterfactual	Average Probability of ≥ 5% weight loss, % (95% CI)	Marginal effect, % (95% CI)	Population Attributable Fraction, % (95% CI)
WMT as observed	17.57 (17.13-18.04)	-	-
No WMT use	17.08 (16.64-17.56)	0.50 (0.48-0.52)	2.81 (2.64-2.96)
No nutrition counselling	17.49 (17.05-17.96)	0.08 (0.06-0.10)	0.47 (0.36-0.58)
No meal replacement	17.51 (17.06-17.98)	0.07 (0.06-0.08)	0.39 (0.34-0.43)
No anti-obesity medications	17.54 (17.09-18.01)	0.04 (0.03-0.05)	0.22 (0.17-0.26)
No bariatric surgery	17.27 (16.83-17.74)	0.30 (0.30-0.31)	1.73 (1.66-1.79)

WMT = Weight Management Treatment

© 2024 Henderson J et al. *JAMA Network Open.*
