# Supplementary material for: Weight Loss Treatment and Longitudinal Weight Change Among Primary Care Patients With Obesity
Source: JAMA Netw Open. 2024 Feb 15;7(2):e2356183. doi: 10.1001/jamanetworkopen.2023.56183 (PMC10870179; doi:10.1001/jamanetworkopen.2023.56183)
Supplement: Supplement 2. — Data Sharing Statement [file jamanetwopen-e2356183-s002.pdf]

Data Sharing Statement

Henderson. Weight Loss Treatment and Longitudinal Weight Change Among Primary Care Patients With Obesity. *JAMA Netw Open*. Published February 15, 2024. doi:10.1001/jamanetworkopen.2023.56183

Data
Data available: No
